# Randomized trial of the feasibility of ED-initiated school-based asthma medication supervision (ED-SAMS)

**DOI:** 10.1186/s40814-021-00913-0

**Published:** 2021-09-27

**Authors:** Lynn B. Gerald, Joe K. Gerald, John M. VanBuren, Ashley Lowe, Cecilia C. Guthrie, Eileen J. Klein, Andrea Morrison, Emily Startup, Kurt Denninghoff

**Affiliations:** 1 University of Arizona, Tucson, AZ USA; 2 University of Utah, Salt Lake City, USA; 3 University of Oklahoma, Norman, USA; 4 Seattle Children’s Hospital, Seattle, USA; 5 Medical College of Wisconsin, Milwaukee, USA; 6 Asthma and Airway Disease Research Center, 1501 N Campbell Avenue, Tucson, AZ 85724 USA

**Keywords:** Asthma, Schools, Pediatric, Pediatric emergency medicine

## Abstract

**Background:**

While using an inhaled corticosteroid (ICS) in the weeks after an ED visit reduces repeat visits, few children receive a needed prescription. Because a prescription may not be filled or used, dispensing ICS at discharge and supervising its use at school could overcome both barriers until follow-up care is established. To assess the feasibility of such an intervention, we conducted a pilot study among elementary-age school children with persistent asthma who were discharged from the ED following an asthma exacerbation.

**Methods:**

Eligible children were randomly assigned to ED-dispensing of ICS with home supervision *or* ED-dispensing of ICS with home *and* school supervision. The primary outcomes were ability to recruit and retain participants, ability to initiate school-supervised medication administration within 5 days of discharge, and participant satisfaction.

**Results:**

Despite identifying 437 potentially eligible children, only 13 (3%) were enrolled with 6 being randomized to the intervention group and 7 to the control group. Eleven (85%) randomized participants completed the 90-day interview (primary outcome) and 8 (62%) completed the 120-day interview (safety endpoint). Four (67%) intervention participants started their school regimen within 5 business days and 2 started within 6 business days.

**Conclusion:**

While our pilot study did not meet its recruitment goal, it did achieve its primary purpose of assessing feasibility before undertaking a larger, more intensive study. Several major recruitment barriers need to be mitigated before EDs can successfully partner with schools to establish supervised ICS treatment.

**Trial registration:**

ClinicalTrials.gov, NCT03952286. Registered 16 May 2019,

## Key messages regarding feasibility

Children who use ICS following an ED visit are half as likely as non-users to experience a repeat visit; however, few children receive a needed prescription. Providing a prescription at discharge does not guarantee that it will be filled by parents or used by the child. Dispensing a controller medication at discharge and supervising its use at school until follow-up care can be arranged could overcome both barriers and potentially reduce ED recidivism. To assess the feasibility of such an intervention, we conducted a pilot study among elementary-age school children with mild-to-moderate asthma who were discharged from the ED following an asthma exacerbation. This study did not meet its recruitment goal and identified several major recruitment barriers that need to be mitigated before EDs can successfully partner with schools to establish supervised ICS treatment.

## Background

Asthma is a common chronic condition of childhood that is associated with substantial morbidity attributable to medication non-adherence [1]. The National Asthma Education and Prevention Program (NAEPP) urges the development of new, more effective programs to address this problem. Schools are a logical setting to deploy such programs because schools are where children congregate, spend much of their day, and are closely monitored [2, 3]. Furthermore, engaging schools that serve minority and low-income students can reach the populations that experience the highest levels of preventable morbidity.

Supervising inhaled corticosteroid (ICS) use at school increases controller medication adherence and reduces episodes of poor asthma control [4]. While doing so can be cost-effective under certain conditions, enrolling students with mild asthma who use health care services infrequently tends to diminish program efficiency [5]. Targeting children who are discharged from the emergency department (ED) following an asthma exacerbation can address this challenge because these children are at higher risk of future exacerbations than their peers [6, 7]. Based on unpublished data, one-third of children treated for an asthma exacerbation in the Pediatric Emergency Care and Research Network (PECARN) experienced a second ED-managed exacerbation within 6 months [8].

Children who use ICS following an ED visit are half as likely as non-users to experience a repeat visit [6]. Dispensing a controller medication at ED discharge and supervising its use at school until follow-up care can be arranged could reduce ED recidivism. To assess the feasibility of such an intervention, we conducted a pilot study among elementary-age school children with mild-to-moderate asthma who were discharged from the ED following an asthma exacerbation.

## Methods

We conducted a randomized trial comparing ED-dispensing of ICS with home supervision (standard of care) with ED-dispensing of ICS with home and school supervision (intervention). The protocol was reviewed and approved by a Data Safety Monitoring Board (DSMB) and a single-site Institutional Review Board (IRB) at the University of Utah. It was also reviewed and approved by each participating school district. This study involved three clinical sites, a data coordinating center, and a clinical coordinating center. During the study, one clinical site was replaced by another after failing to secure project approval from its local school district.

Children were eligible if they were 6–12 years of age, were discharged home after successful treatment for an asthma exacerbation, and were enrolled full-time in a participating school district. Within the ED, care was provided at the sole discretion of the primary ED provider including all tests, procedures, and treatments deemed appropriate to improve pulmonary function and safely discharge the participant home. At discharge, participants were randomly assigned in a 1:1 ratio to either: ED-dispensing of ICS with home supervision (standard of care) *or* ED-dispensing of ICS with home *and* school supervision (intervention). All participants received oral prednisolone to achieve a daily dose of 2 mg/kg not to exceed 40 mg per day for 5 days or its equivalent. For home use, all participants also received a budesonide dry powder inhaler (360 μg once-daily) and an albuterol sulfate metered dose inhaler with spacer (as needed). Intervention participants had one additional inhaler of each type and a spacer couriered to the child’s school health office where its use was supervised on school days; parents continued to supervise administration on weekends, holidays, and school absences. The randomization protocol was determined by the Data Coordinating Center using a block permutation design to ensure balanced randomization given the small sample sizes required at each site.

### Primary outcome and sample size

The primary outcomes were ability to recruit and retain participants, ability to initiate school-supervised medication administration within a goal time frame of 5 days and participant satisfaction. The pilot trial was designed to inform a larger multi-center clinical trial that capitalizes on the full experience, infrastructure, and reach of the 18 PECARN sites. The primary outcome for the larger clinical trial was expected to be 90-day ED recidivism. While the primary purpose of the ED-SAMS pilot was to determine the feasibility and acceptability of the proposed intervention, we also planned to estimate the confidence interval of the intervention’s effect size. We were aware that small samples such as this one yield large standard errors and wide confidence intervals. However, we believed the pilot data would be informative with regard to the intervention’s potential impact and whether future trials are likely to hold promise. Furthermore, the results can inform future analytic approaches and suggest a plausible range of potential effect sizes. As such, the focus was not on significance testing but rather on estimating effect sizes and corresponding confidence intervals. Randomization of 90 patients would allow estimation of consent and approach rates to within 8% (half-width of 95% confidence interval).

Data are presented using traditional summary statistics. Categorical variables are presented using counts and frequencies. Continuous variables are presented using means and standard deviations. Due to small sample sizes, statistical inferences were not performed comparing the two groups.

No interim analyses were planned. This was a pilot study involving recruitment over a short period of time. There would be insufficient time or data to analyze, present, and then act upon.

## Results

**Fig. 1:**
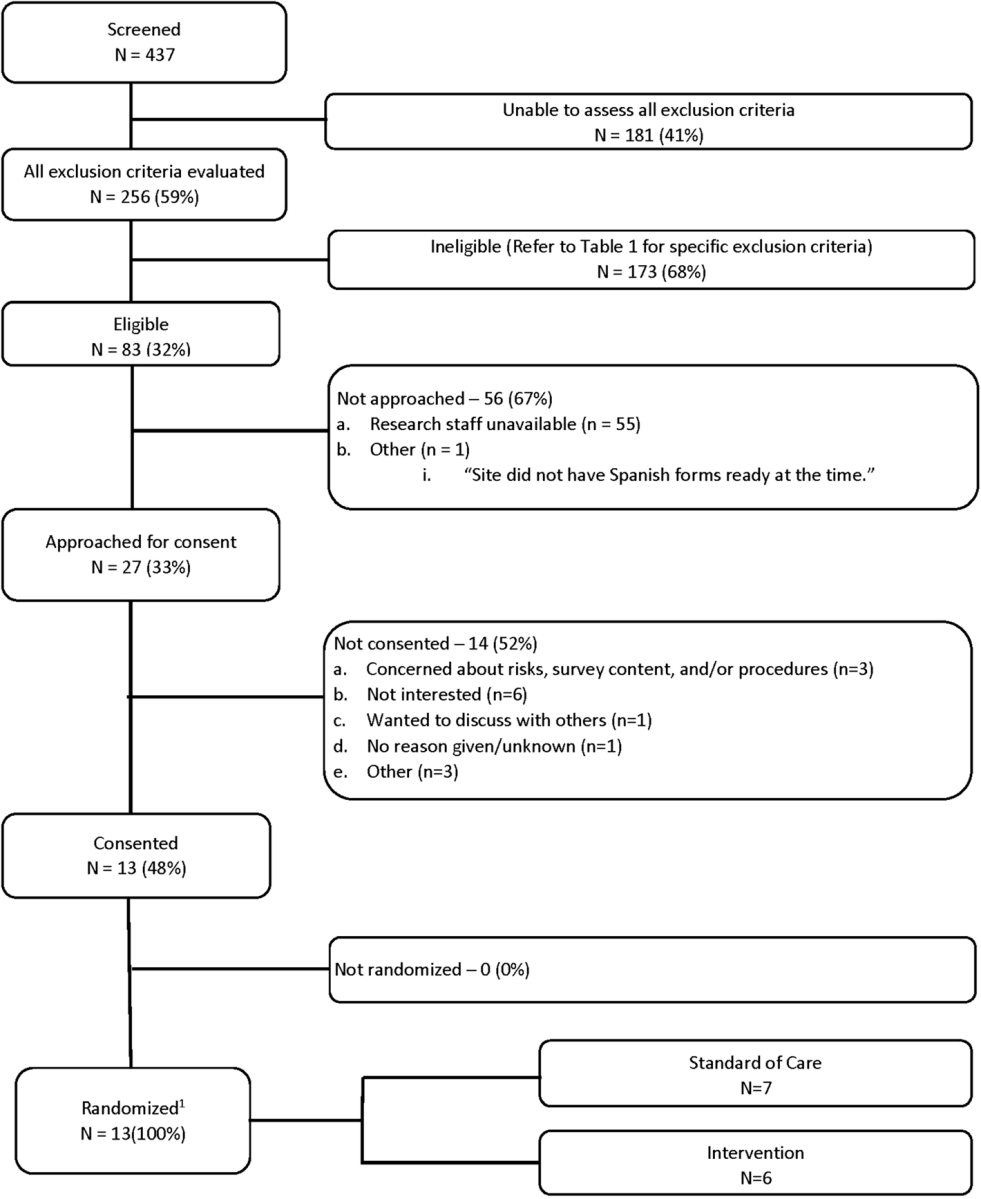
ED-SAMS Consort Diagram ^1^Patients could be ineligible due to more than one exclusion reason

Based on age and presenting complaint, 437 children were potentially eligible but 181 (41%) were not further screened because a research coordinator was not present at the time of the visit to assess all of the exclusion criteria. Of the 256 who were fully screened, 173 (68%) were excluded: 75 did not attend a participating school, 72 would have required a step-down of their usual controller regimen, and 26 were ineligible for other reasons (Fig. 1 and Table 1). Of the 83 who were eligible, 56 (67%) were not approached because a research coordinator, while present, was otherwise engaged with a competing research study. Of the 27 eligible participants who were approached, 13 (48%) enrolled with 6 being randomized to the intervention group, and 7 to the standard of care group.

**Table 1:** Summary of exclusion reasons for ineligible subjects^1^

	***N*** (%) (***N*** = 173)
**Does the patient attend a non-participating school?**	75 (43.4%)
**Among patients who attend a participating school (or had missing value for that exclusion criteria)**	98 (56.6%)
**Does the study medication represent a step-down in asthma therapy in the judgement of the ED physician?**	72 (73.5%)
**Does the patient have a history of using greater than or equal to 2 controller medications for asthma in the past 30 days?**	19 (19.4%)
**Is the patient hospitalized?**	11 (11.2%)
**Is the patient or their parent/guardian non-English or non-Spanish speaking?**	9 (9.2%)
**Has the patient had greater than or equal to 2 hospitalizations for asthma in the past year?**	6 (6.1%)
**Is the patient enrolled in another research study?**	2 (2.0%)
**Does the patient attend a participating school less than 5×/week?**	0 (0%)
**Has the patient had any ICU admissions for asthma in the past year?**	0 (0%)
**The parent does not have a cell phone.**	0 (0%)
**The parent cannot send and receive text messages from their cell phone.**	0 (0%)

^1^Patients could be ineligible due to more than one exclusion reason

Eleven (85%) randomized participants completed the 90-day interview (primary outcome) and 8 (62%) completed the 120-day interview (safety endpoint). Four (67%) intervention participants started their school regimen within the study goal of 5 business days and the other 2 started within 6 business days (Table 2). While there were too few participants too permit meaningful comparisons, 1 (17%) intervention participant and 2 (29%) control participants experienced a repeat asthma-related ED visit within 90 days.

**Table 2:** Demographics and follow-up by arm

		Randomization arm	
	Overall (*N* = 13)	Arm 1: Standard of care (*N* = 7)	Arm 2: Intervention (*N* = 6)
**Age at randomization**	7.9 (1.57)	8.4 (1.86)	7.2 (0.89)
**School grade**			
Kindergarten	3 (23.1%)	1 (14.3%)	2 (33.3%)
1st	4 (30.8%)	2 (28.6%)	2 (33.3%)
2nd	3 (23.1%)	1 (14.3%)	2 (33.3%)
3rd	1 (7.7%)	1 (14.3%)	0 (0.0%)
4th	1 (7.7%)	1 (14.3%)	0 (0.0%)
6th	1 (7.7%)	1 (14.3%)	0 (0.0%)
**Sex**			
Male	7 (53.8%)	4 (57.1%)	3 (50.0%)
Female	6 (46.2%)	3 (42.9%)	3 (50.0%)
**Race**			
Black or African American	9 (69.2%)	4 (57.1%)	5 (83.3%)
White	2 (15.4%)	1 (14.3%)	1 (16.7%)
Multiple	1 (7.7%)	1 (14.3%)	0 (0.0%)
Unknown	1 (7.7%)	1 (14.3%)	0 (0.0%)
**Ethnicity**			
Hispanic or Latino	2 (15.4%)	1 (14.3%)	1 (16.7%)
Not Hispanic or Latino	10 (76.9%)	5 (71.4%)	5 (83.3%)
Unknown or Not reported	1 (7.7%)	1 (14.3%)	0 (0.0%)
**Parent/guardians preferred language**			
English	13 (100.0%)	7 (100.0%)	6 (100.0%)
**Student qualifies for free/reduced school lunch program**			
Yes	10 (76.9%)	5 (71.4%)	5 (83.3%)
No	1 (7.7%)	1 (14.3%)	0 (0.0%)
**Within 5 weekdays days from randomization to school receiving medication**			
Yes	–	–	4 (66.7%)
No	–	–	2 (33.3%)
**Completed 90-day interview** (primary outcome)			
Yes	11 (84.6%)	6 (85.7%)	5 (83.3%)
No	2 (15.4%)	1 (14.3%)	1 (16.7%)
**Completed 120-day interview** (safety outcome)			
Yes	8 (61.5%)	6 (85.7%)	2 (33.3%)
No	5 (38.5%)	1 (14.3%)	4 (66.7%)
**Experienced repeat asthma-related ED visit < 90 days**			
Yes	3 (23.1%)	2 (28.6%)	1 (16.7%)
No	10 (76.9%)	5 (71.4%)	5 (83.3%)

## Discussion

Enrolling only 13 participants in total, our recruitment effort fell far short of its 90-participant goal, 5 children per site per month. While an early medication acquisition challenge led us to shorten our recruitment window from 9 to 6 months, recruitment would have still fallen far short of our goal with the longer window. Because only 3% of 437 potentially eligible children ultimately enrolled, recruitment was the study’s primary failure mode and most important obstacle to feasibility. Several factors accounted for a disproportionate share of screen failures.

Having a research coordinator present and available during the child’s ED visit was a major barrier as 237 (54%) of the 437 potentially eligible participants were excluded for these two reasons. If these children could have been approached with a similar enrollment yield as those who were approached when a coordinator was available, then another 55 children might have participated. Even so, the study would have still fallen 21 participants short of its goal. Having a research coordinator present during the treatment window (i.e., visit occurred after hours) was a greater barrier than ensuring one was available to approach the child’s family (i.e., coordinator engaged in other job duties), 181 versus 56 excluded participants, respectively. Ensuring 24 h, 7 days per week coverage is prohibitively expensive for all but the largest projects. Knowing this, we had hoped our pilot study could “piggyback” on each site’s dedicated full-time PECARN research coordinator. The expectation was patient volume would be large enough during the coordinator’s typical work hours to offset missed opportunities due to competing responsibilities. Given the financial constraints of the R34 mechanism, this challenge will be difficult to overcome without a changing to a more generous funding mechanism or modifying our recruitment strategy.

Next-day, telephone recruitment after the child’s discharge could have potentially supplemented our real-time, in-person recruitment by reaching families of children who passed the initial screen but who were not subsequently approached. Undoubtedly, some eligible children might still have been missed, or some families might have declined to participate because doing so might have disrupted their already established discharge treatment plan. Nevertheless, next-day, telephone recruitment could have presented coordinators with more opportunities to recruit during normal business hours when ED volume was lower and when greater flexibility existed to avoid competing responsibilities.

Even if coordinators had been more readily available, 30% of children would have still been excluded because they attended a non-participating school district. While a lesser challenge than coordinator availability, this factor was still a significant recruitment barrier. Research linking the traditional health system (EDs) with community partners (schools) can impose unique challenges that impact study design and recruitment. One of the most important of these challenges is the regulatory measures intended to ensure the safety of human subjects. IRB approval or its equivalent is required from the university, the school district, and frequently, individual schools (e.g., consent of the principal). While necessary and appropriate, these activities are time-consuming and administratively complex as each school district has its own regulatory system.

Typically, the regulatory burdens imposed by school collaborations can be overcome with adequate time and resources, especially if researchers can leverage established relationships from past collaborations. However, this was PECARN’s first school-based project so none of the clinical sites had established relationships to help them navigate the schools’ regulatory processes. Knowing this, clinical sites were only required to obtain approval from the largest school district within their catchment area. Because each clinical site was located in a large metropolitan area, each could engage a large school district serving tens of thousands of students. Despite our success establishing new collaborations, aggregate student enrollment in the smaller, non-participating districts still invalidated a substantial portion of children who would have been otherwise eligible to participate.

While these regulatory burdens imperiled our research project, they would not necessarily impair real-world collaborations between EDs and schools. In clinical practice, many physicians already designate medication administration to occur at school (e.g., amphetamine treatment for attention deficit hyperactivity disorder). Existing state laws facilitate this practice by requiring schools to have protocols to safely administer prescription and over-the-counter medications to students. Accordingly, schools have standard practices for administering prescribed medications [9]. For example, most require parents, and sometimes physicians, to sign medication administration forms instructing schools on how and when to administer the medication and authorizing them do so. Assuming parents can overcome the logistical barriers inherent in obtaining medication for school use (e.g., a second inhaler just for school use), medication administration itself is feasible under most circumstances. In fact, 5% of children in the USA receive at least one prescription medication at school daily [9].

The last recruitment barrier was the study’s decision to use a standardized single-drug controller regimen that was FDA-approved for once-daily use. Approximately 30% of screened children who attended a participating school were excluded because the treating ED physician judged that once-daily ICS monotherapy would result in a step-down from the child’s prior controller regimen. Interestingly, only 1 in 5 children who attended a participating school were excluded because they reported using a combination inhaler (e.g., an inhaled corticosteroid plus a long-acting beta-agonist, ICS/LABA). While we considered replicating the child’s usual controller regimen to expand eligibility, doing so would have increased medication acquisition costs, precluded once-daily use in many instances, and potentially confounded study outcomes owing to differing treatment regimens.

The underlying assumption justifying school-supervised asthma therapy is that higher adherence to a potentially less efficacious controller regimen at school (e.g., once-daily ICS monotherapy) is ultimately more effective than obtaining lower adherence to a potentially more efficacious controller regimen at home (e.g., twice daily ICS/LABA combination therapy). Because budesonide inhalation powder was approved by the Food and Drug Administration (FDA) for once-daily administration, it was an obvious choice for a standardized school-supervised regimen in the absence of an ICS/LABA combination with a similar FDA approval. Decisions like this one highlight the trade-offs that often occur when attempting to balance efficacy-centric designs, asking how well interventions work under ideal conditions, with effectiveness-centric designs asking how well they work under real-world conditions [10].

Because this pilot was intended to replicate real-world conditions, in our original protocol, ED physicians were expected to prescribe, not dispense, budesonide inhalation powder at discharge to control participants. However, budesonide inhalation powder was not on the Medicaid-approved drug formulary at some clinical sites so many patients would not have been able to fill their prescription. To ensure every child could obtain medication, we changed the prescribing arm to a dispensing arm and simply purchased and dispensed budesonide dry powder inhalers to everyone. This change resulted in a 3-month recruitment delay while we obtained DSMB approval of this change. In addition to creating a delay, this change may have also impaired recruitment because replicating the child’s existing controller regimen would have avoided concerns related to stepping down care.

## Conclusions

While unsuccessful in meeting its recruitment goal, our pilot study did achieve its primary purpose of assessing feasibility before undertaking a larger, more resource intensive study. Asthma research linking EDs with schools will not be successful until the previously mentioned recruitment barriers can be rectified or at least mitigated. Our previous successful efforts establishing school-supervised administration of asthma controller medications in large, public school districts offers few easy solutions [4, 11]. While recruitment fell short of our goal, this pilot project enhanced PECARN’s capacity to undertake future community-based efforts by establishing new school collaborators and identifying areas needing improvement. Leveraging ED–school collaborations to improve controller medication adherence and reduce ED recidivism might still be successful if recruitment barriers can be overcome. After all, half of eligible families who were asked to participate enrolled with 85% of them completing the 90-day follow-up interview. Furthermore, all intervention participants began their school controller in a timely fashion. Therefore, a larger trial which had 24/7 ED coverage or used next-day recruitment following the child’s ED discharge could be successful. Future trials could also work to enroll more school districts.

## Data Availability

The datasets used and/or analyzed during the current study are available from the corresponding author on reasonable request.

## Abbreviations

DSMB: Data Safety and Monitoring Board
ED: Emergency department
ICS: Inhaled corticosteroids
IRB: Institutional Review Board
NAEPP: National Asthma Education and Prevention Program
PECARN: Pediatric Emergency Care and Research Network
